# Progestin-Primed Ovarian Stimulation Protocol for Patients With Endometrioma

**DOI:** 10.3389/fendo.2022.798434

**Published:** 2022-04-28

**Authors:** Ai-Min Yang, Teng-Fei Feng, Yan Han, Zhi-Ming Zhao, Wei Wang, Yi-Zhuo Wang, Xiao-Qi Zuo, Xiuhua Xu, Bao-Jun Shi, Lipeng Li, Gui-Min Hao, Na Cui

**Affiliations:** ^1^ Hebei Key Laboratory of Infertility and Genetics, Hebei Clinical Research Center for Birth Defects, Department of Reproductive Medicine, Second Hospital of Hebei Medical University, Shijiazhuang, China; ^2^ Cardiovascular Platform, Institute of Health and Disease, Hebei Medical University, Shijiazhuang, China; ^3^ Hebei Key Laboratory of Infertility and Genetics, Hebei Clinical Research Center for Birth Defects, Department of Pediatric Surgery, Second Hospital of Hebei Medical University, Shijiazhuang, China

**Keywords:** *in vitro* fertilization-embryo transfer (IVF-EF), progestin-primed ovarian stimulation (PPOS), ultra-long GnRH agonist protocol, endometrioma, live birth

## Abstract

**Objective:**

To evaluate the pregnancy outcomes of progestin-primed ovarian stimulation (PPOS) protocol for patients with endometrioma underwent *in vitro* fertilization/intra-cytoplasmic sperm injection embryo transfer (IVF/ICSI-ET).

**Design:**

Observational retrospective cohort study.

**Setting:**

University affiliated reproductive center.

**Study Participants:**

605 infertile patients with endometrioma underwent IVF/ICSI-ET from January 2016 to March 2021 were included in this study.

**Methods:**

Multivariable logistic regression analyses were conducted to determine the independent effect of controlled ovarian stimulation (COS) protocols on reproductive outcomes of first embryo transfer (ET) cycles. The live birth was primary outcome, the implantation rate, biochemical pregnancy, clinical pregnancy and ongoing pregnancy were secondary outcomes.

**Results:**

Compared to PPOS protocol, the probability of implantation showed no significant difference with ultra-long gonadotrophin-releasing hormone agonist (GnRHa) protocol and gonadotrophin-releasing hormone antagonist (GnRHant) protocol (OR 1.7, 95% CI 0.9-3.1, OR 1.2, 95% CI 0.7-2.1, respectively). The PPOS protocol was correlated with a significantly lower biochemical pregnancy and clinical pregnancy than ultra-long GnRHa protocol in the multivariable logistic regression analysis (OR 2.3, 95% CI 1.1-4.9, OR 2.4, 95% CI 1.1-5.3, respectively). However, there was no significant difference in terms of biochemical pregnancy, clinical pregnancy and ongoing pregnancy between PPOS and GnRHant protocol (OR 1.4, 95% CI 0.7-2.7, OR 1.3, 95% CI 0.7-2.4, OR 1.1, 95% CI 0.6-2.3, respectively). In addition, compared to PPOS protocol, ultra-long GnRHa protocol and GnRHant protocol demonstrated no statistical difference in ongoing pregnancy (OR 2.0, 95% CI 0.9-4.5, OR 2.1, 95% CI 0.6-2.3, respectively). Notably, the ultra-long GnRHa protocol was associated with a significant higher probability of live birth than PPOS protocol both in crude analysis and multivariable logistic regression analysis (OR 2.6, 95% CI 1.3-5.1, OR 2.5, 95% CI 1.1-5.7, respectively). Nevertheless, no statistical difference was found in live birth between PPOS and GnRHant protocol either in crude analysis and multivariable logistic regression analysis (OR1.2, 95% CI 0.6-2.3, OR 1.2, 95% CI 0.6-2.5, respectively).

**Conclusions:**

Based on the reproductive outcomes of the first ET cycles in patients with endometrioma, PPOS protocol may associated with inferior reproductive outcomes in terms of biochemical pregnancy, clinical pregnancy and live birth than ultra-long GnRHa protocol. However, there was no significant difference in implantation rate, clinical pregnancy, ongoing pregnancy and live birth between PPOS and GnRHant protocol.

## Introduction

Endometriosis is a common inflammatory disease which is known to be associated with progressive dysmenorrhea, chronic pelvic pain and infertility (1). Endometriosis is affecting up to 10%~15% (2) of reproductive-aged women and 40%~70% of patients with pain and infertility (3). Endometrioma is manifested by a chocolate fluid-containing cyst located inside the ovary and very common form of pelivc endometriosis. The prevalence of ovarian endometrioma was reported to be 17~44% among women with endometriosis (2). Endometrioma is very common in patients scheduled for IVF/ICSI-ET, accounting for up to 20~40% (4).

The reason why endometrial cells implant in the ovaries has not yet been fully understood. Endometriotic ovarian cysts can be easily distinguished from other ovarian cysts by specialized transvaginal ultrasound without laparoscopic and histological confirmation (5). Preoperative evaluation by ultrasound allows clinicians to provide appropriate infertility counseling and treatment to patients with endometrioma (6). The exact pathophysiology of endometrioma in infertility also remains under debate. Studies have proposed that tubo-ovarian anatomy might be directly distorted (7), while inflammatory (8) and oxidative damage (9) to the oocytes could be indirectly invoked (10). The detrimental impact of endometrioma on IVF/ICSI-ET treatment included various aspects: the difficulties in follicular aspiration, the risk of developing ovarian abscess and pelvic infection or endometrioma rupture after oocyte retrieval. Treatment options for endometrioma include expectant management, medical and/or surgical treatment and IVF-ET. Emerging evidence suggested that patients with endometrioma should not systematically remove endometrioma before IVF procedure to reduce time to live birth, avoid potential surgical complications and limit costs (6, 11). Moreover, although the number of oocytes retrieved were fewer in women with endometrioma due to the diminished ovarian reserve, studies suggested similar clinical pregnancy and live birth rates with patients without endometrioma (11, 12).

For patients with endometriosis, the optimal controlled ovarian stimulation (COS) is still controversial. Many COS strategies have been offered, but no compelling advantage of one COS protocol over another has been established. Up to now, most of the published data suggested that patients with endometriosis should be treated with prolonged GnRHa protocol, mainly due to the suppression effect of GnRHa on ectopic endometrial tissue (13, 14). However, A recent review suggested that GnRH antagonist or PPOS protocol could be more appropriate than prolonged suppression protocol for endometriosis (15). Sparse studies focused on the appropriate protocol in cases of endometrioma. The PPOS protocol involves pituitary suppression by oral progestins started simultaneously with the gonadotrophins, has been widely used in recent years. A Study published in 2020 has showed that PPOS protocol could be a choice for fertility preservation in women with endometriosis (16). What about patients with endometrioma? In the current study, we retrospectively analyzed data from patients with endometrioma undergoing IVF/ICSI-ET and completed their first embryo transfer cycles. The reproductive outcomes of PPOS protocol were compared with ultra-long GnRHa and GnRHant protocol.

## Materials and Methods

### Study Participants and Design

The current study is a real-world observational cohort study. The ethics committee of the Second Hospital of Hebei Medical University approved the current study. All participants allowed the use of their medical records and signed written informed consents before the treatment. Patients were aged 20~44 years. The exclusion criteria included cycles using gametes from donors, fertility preservation cycles and pre-implantation genetic testing (PGT) cycles. Patients with systemic lupus erythematosus, antiphospholipid syndrome (APS), known mullerian duct anomalies, intrauterine adhesion, and untreated hydrosalpinx were also excluded. After all exclusions, 605 patients with diagnosis of endometrioma underwent IVF/ICSI-ET between January 2016 and March 2021 were enrolled. All the patients completed COS procedure and 63 patients canceled because of no viable embryos. 492 patients underwent their first ET cycles. The reproductive outcomes of their first ET cycles originating from the three COS protocols were recorded and analyzed.

Endometrioma was diagnosed through imaging modalities (6), surgical (laparoscopic or laparotomy) removal and histological confirmation or aspiration of chocolate-colored fluid at the time of oocytes retrieval. Moreover, the diagnosis of endometrioma by ultrasound had to be examined on at least two menstrual cycles apart (12).

### Ovarian Stimulation Regimen

Patients underwent transvaginal ultrasound and baseline hormone test before ovarian stimulation and those with all follicle diameter <10 mm, FSH <14mIU/ml, and thickness of endometrium < 6mm began to COS. The starting dose of gonadotrophin was determined based on maternal age, body mass index (BMI) and ovarian reserve. The recombinant/urinary FSH (Gonal-F, Merck Serono, Italy, Urofolitropin for injection, Lizhu Pharmaceutical, China) alone or in combination with hMG (Menotropins, Lizhu Pharmaceutical, China) with total doses 150~300IU/day were used for ovarian stimulation. Follicular growth was monitored by transvaginal ultrasound and hormone measurements and the doses of gonadotrophin was adjusted accordingly. All of the patients underwent the freeze-all strategy in PPOS protocol. In ultra-long GnRHa protocol and GnRHant protocol, the freeze-all strategy was implemented in patients with high risk of ovarian hyperstimulation syndrome (OHSS), high progesterone levels on the trigger day and elevated serum CA-125 level.

### PPOS Protocol

COS was started on 2~4 days of menstrual cycle. Medroxyprogesterone Acetate (MPA) (10 mg/day, Zhejiang Xianju Pharmaceutical Co., China) or Duphaston (20 mg/d; Abbott Biologicals B.V., Netherlands) was administrated with gonadotrophin from the initial of COS and continued up to the trigger day. As soon as at least three follicles reached the diameter of ≥18mm, the maturation of follicles was triggered by Decapeptyl (0.1 mg; Ferring Pharmaceuticals Ltd., Saint-Prex, Switzerland) and human chorionic gonadotropin (hCG) (1000 IU; Lizhu Pharmaceutical Trading Co., China) (17). Oocyte aspiration was performed 35.5~36.5 hours after trigger.

### Ultra-Long GnRH Agonist Protocol

Ultra-long GnRH agonist protocol has been described previously (7). Patients received a depot injection of triptorelin acetate (3.75mg; Ferring Pharmaceuticals, Kiel, Germany) 28~30 days before ovarian stimulation on 2~4 days of menstrual cycle and COS started 28~35 days after the last injection. When at least three dominant follicles reached the diameter of 18~20mm, recombinant hCG (250ug; Merck Serono, Coinsins, Switzerland) was injection intramuscularly. The oocytes aspiration was performed 36.5~37.5 hours after trigger.

### The GnRH-Antagonist Protocol

Flexible GnRHant protocol was used in this study. When the leading follicle was observed to be ≥14 mm in diameter or estradiol concentration reached ≥400pg/ml, GnRH-antagonist (0.25mg/day, Merck Serono, Coinsins, Switzerland) injection was started until the trigger day. When three dominant follicles reached 17 mm in diameter, the final maturation of oocytes was induced by recombinant hCG (250ug; Merck Serono, Coinsins, Switzerland). The oocyte aspiration was performed 35.5~36.5 hours after triggering.

### Embryo Culture and Frozen-Thawed Embryo Transfer

Sperms were prepared by swim-up. Fertilization was carried out *in vitro* by either IVF or ICSI according to the sperm parameters (18). The Istanbul consensus scoring system was applied for the scoring of the embryos (19). One or two rating of good or fair cleavage-stage embryos were selected to transfer or cryopreserved by vitrification on the third day after oocyte retrieval. All of the remaining embryos were cultured to day 5~7 when they reached the blastocyst stage. Blastocysts which rating of good or fair were frozen during this stage. In fresh cycles, vaginal administration of progesterone gel (Crinone, Merck Serono, Watford, UK) was used from the morning of oocyte retrieval day for luteal support. In frozen/thawed embryo transfer (FET) cycles, endometrial preparation regimen and luteal support were performed as previously described (20). Briefly, natural cycle, letrozole induced ovulation, hormone replacement therapy (HRT) with or without downregulation were used for endometrial preparation in FET cycles. A maximum of 2 embryos were transferred per cycle. For natural and letrozole induced ovulation protocol, oral dydrogesterone (10mg; Duphaston, Abbott, OLST, Netherlands) twice a day was used for luteal support. For HRT protocol, oral dydrogesterone 10 mg three times a day and vaginal progesterone gel (90mg; Crinone, Merck Serono, Watford, UK) once a day were used for luteal support. If pregnancy was detected, luteal support was continued until 8 gestation weeks. Embryos with highest morphological scores were preferentially transferred in their first embryo transfer cycles.

### Outcome Measures

Reproductive outcomes of the first ET cycles of the patients were analyzed in this study. The primary outcome was live birth (at least an alive birth after 28 gestational weeks) per embryo transfer. The secondary outcomes included implantation rate, clinical pregnancy, biochemical pregnancy and ongoing pregnancy per embryo transfer. Implantation rate was calculated as the number of gestational sacs observed by ultrasound divided by the number of embryos transfer (21). Biochemical pregnancy was defined as serum hCG level ≥5.3mIU/mL 12 days after embryo transfer. Clinical pregnancy was defined as observation of an intrauterine gestational sac *via* transvaginal ultrasound or villous tissue confirmed by histology test. Early miscarriage was defined as spontaneous clinical abortion before 12 weeks. Ongoing pregnancy was defined as a viable intrauterine pregnancy of at least 12 weeks duration confirmed on an ultrasound scan. Late miscarriage was defined as spontaneous abortion between 12 and 28 gestational weeks.

### Statistical Methods

Patients’ demographic characteristics, IVF-ET cycle-specific characteristics, and reproductive outcomes were assessed among groups. The normality of continuous variables was tested by the Kolmogorov-Smirnova test and Q-Q plots. The normally distributed data were presented as means (standard deviations) (SDs). The non-normally distributed data were presented as median (25^th^ percentile-75th percentile). The one-way ANOVA test with Bonferroni’s adjustment were applied for the comparisons of normally distributed data. The Kruskal-Wllis H test with Bonferroni’s adjustment was applied for the comparisons of non-normally distributed continuous data. Data was presented as number (percentage) for categorical variables. Comparisons of categorical variables was performed by Chi square test or Fisher’s exact probabilities. Generalized estimating equation (GEE) analysis was conducted to determine the difference in odds ratio for implantation rate among the three groups. Multivariable logistic regression analysis was conducted to determine the independent effect of COS protocols on reproductive outcomes (implantation rate, biochemical pregnancy, clinical pregnancy, ongoing pregnancy and live birth). This analysis included all participants who completed their first ET cycles. The covariates were selected on the basis of their associations with the reproductive outcomes of interest or a change in effect estimate of more than 10% (22). The following seven covariates were added to the main effect: maternal age, maternal BMI, total antral follicle count (AFC), adenomyosis, number of transferred embryos (1 versus 2), fertilization method. The predictive ability of binary logistic regression models often rely on an Events Per Variable criterion (EPV), notably EPV≥10, to determine the minimal sample size required (23). We included 8 variables to the multivariable logistic regression models in our study and all the numbers of the events (implantation, biochemical pregnancy, clinical pregnancy, ongoing pregnancy and live birth) met the requirement of EPV (more than 80 cases). All analyses were performed using SPSS 20.0 or Empower (R) (www.empowerstats.com, Boston MA) and R (http://www.R-project.org). *P* < 0.05 was considered as statistically significant.

## Results

### Study Participants

**Figure 1:**
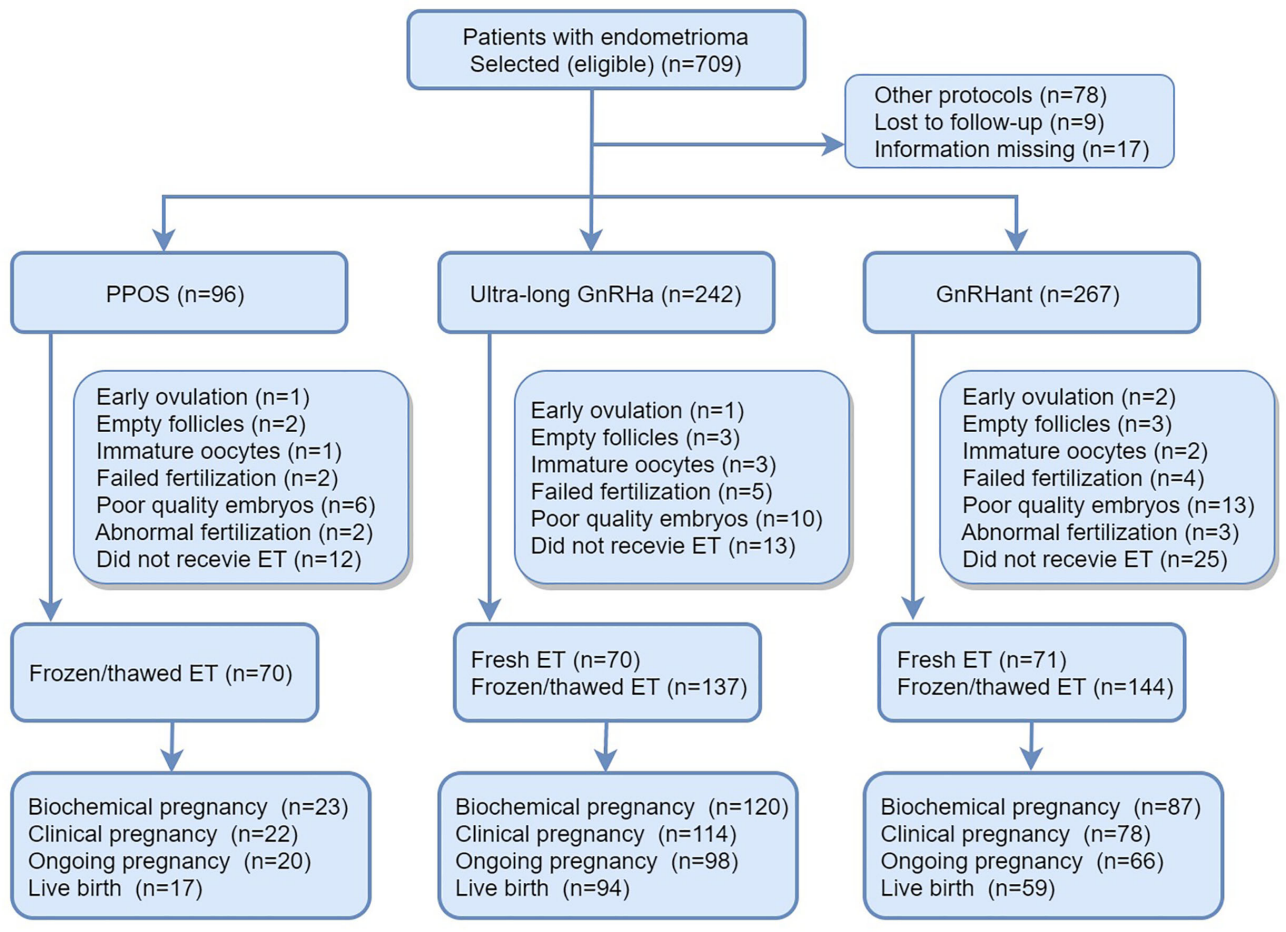
Details of flowchart of participants and main reproductive outcomes of the enrolled patients. PPOS, progestin-primed ovarian stimulation; GnRHa, gonadotrophin-releasing hormone agonist; GnRHant, gonadotrophin-releasing hormone antagonist; ET, embryo transfer.

Details of flowchart of participants and main reproductive outcomes of the enrolled patients were provided in **Figure 1**. In brief, a total of 709 patients with endometrioma underwent COS and 104 patients were excluded according to exclusion criteria. 70 patients canceled because of premature ovulation, empty follicles, immature oocyte, failed fertilization, abnormal fertilization or embryos with poor quality. 50 patients didn’t receive embryo transfer by the research closing date. At the end, 492 patients completed their first ET cycles, which constituted the population of the main analysis. In the PPOS protocol, 70 patients received FET. In the ultra-long protocol, 70 patients received fresh ET and 137 patients received FET. In GnRHant protocol, 71 patients received fresh ET and 144 patients received FET.

Baseline characteristics of the participants in the three groups were shown in **Table 1**. The maternal age in the PPOS group was higher than ultra-long GnRHa group but similar with GnRant group: 34.0 (29.0-37.0) versus 30.0 (28.0-33.0) versus 33.0 (29.5-36.0), *P <*0.001. The total AFC in PPOS group was lower compared with ultra-long GnRHa group but showed no difference with GnRHant group: 5.0 (3.0-8.2) versus 11.0 (7.0-15.0) versus 6.0 (4.0-9.0), *P <*0.001. In addition, male age, previous failed cycles, baseline follicle-stimulating hormone (FSH) were different among the three groups (*P*<0.05). However, maternal BMI 22.4 (3.7) versus 22.9 (3.7) versus 23.1 (4.4), *P* = 0.092 and duration of infertility 3.0 (2.0-6.0) versus 3.0 (2.0-5.0) versus 3.0 (2.0-6.0), *P* = 0.0.158 demonstrated no statistical difference among the three groups. Moreover, the percentage of patients co-exist with adenomyosis was similar among the groups: 20.8% versus 14.0% versus 14.2%, *P*=0.246.

**Table 1:** Baseline characteristics of the participants in the three groups.

	PPOS	Ultra-long GnRHa	GnRHant	*P*
Cycles	96	242	267	
Maternal age (years)	34.0 (29.0-37.0)^a^	30.0 (28.0-33.0)	33.0 (29.5-36.0)^a^	<0.001
Maternal BMI (kg/m^2^)	22.4 (3.7)	22.9 (3.7)	23.1 (4.4)	0.429
Maternal education, n (%)				0.002
Less than senior high school	11 (11.5%)	48 (19.8%)	43 (16.1%)	
Senior high school	10 (10.4%)^b^	44 (18.2%)	69 (25.8%)^b^	
College or above	75 (78.1%)^ab^	150 (62.0%)^a^	155 (58.1%)^b^	
Male age (years)	34.0 (29.0-37.0)^a^	31.0 (28.0-33.0)^a^	33.0 (29.0-38.0)	<0.001
Male BMI (kg/m^2^)	25.3 (3.4)	25.6 (3.9)	26.2 (3.9)	0.141
Total AFC	5.0 (3.0-8.2)^a^	11.0 (7.0-15.0)^a^	6.0 (4.0-9.0)	<0.001
Adenomyosis	20 (20.8%)	34 (14.0%)	38 (14.2%)	0.246
Duration of infertility (years)	3.0 (2.0-6.0)	3.0 (2.0-5.0)	3.0 (2.0-6.0)	0.158
Type of infertility, n (%)				0.244
Primary infertility	65 (67.7%)	167 (69.0%)	166 (62.2%)	
Secondary infertility	31 (32.3%)	75 (31.0%)	101 (37.8%)	
Baseline FSH	9.1 (7.3-12.3)^ab^	7.1 (5.9-8.4)^a^	8.1 (6.4-10.8)^b^	<0.001
Baseline AMH	1.0 (0.6-2.3)^a^	2.6 (1.7-4.1)^a^	1.4 (0.7-2.3)	<0.001
Baseline PRL	13.1(6.7)	15.2 (9.0)	14.1 (7.6)	0.195
Previous failed cycles, n (%)				<0.001
0	23 (24.0%)^a^	143 (59.1%)^a^	87 (32.6%)	
1	46 (47.9%)^a^	82 (33.9%)^a^	130 (48.7%)	
≥ 2	27 (28.1%)^a^	17 (7.0%)^a^	50 (18.7%)	
Gravidity, n (%)				0.108
0	63 (65.6%)	163 (67.4%)	153 (57.3%)	
1	23 (24.0%)	50 (20.7%)	66 (24.7%)	

Categorical variables were presented as n (%) and calculated by Pearson’s chi square test or Fisher’s exact test as appropriate. Continuous data was presented as mean ± SD (normally distributed) and median (25th percentile-75th percentile) (non-normally distributed). The one-way ANOVA test or Kruskal-Wllis H test with Bonferroni’s adjustment was used for the comparisons of continuous variables among groups.

PPOS, progestin-primed ovarian stimulation; GnRHa, gonadotrophin-releasing hormone agonist; GnRHant, gonadotrophin-releasing hormone antagonist; BMI, body mass index; AFC, antral follicle count; FSH, follicle-stimulating hormone; AMH, anti-Müllerian hormone; PRL, prolactin.

a Comparison between PPOS and GnRHa, P < 0.05.

b Comparison between PPOS and GnRHant, P < 0.05.

### Ovarian Stimulation Characteristics

**Table 2** showed the differences in characteristics of ovarian stimulation among the study groups. The days of stimulation were different among the three groups: 10.0 (8.0-11.0) versus 11.0 (10.0-12.0) versus 9.0 (8.0-11.0), *P*<0.001. The total of gonadotropin dose in the PPOS group was lower than ultra-long GnRHa group, but similar with GnRHant protocol: 2662.5 (2100.0-3300.0) versus 2700.0 (2250.0-3440.6) versus 2400.0 (1846.9-3075.0), *P*=0.039. There was no difference in the cancellation rate among the three groups (14.6% versus 9.1% versus 10.1%, *P*=0.322). The number of oocytes obtained was lower in the PPOS group compared with ultra-long GnRHa group, but were comparable with GnRHant group: 4.0 (2.8-7.0) versus 11.0 (6.0-19.0) versus 4.0 (2.5-7.0), *P*<0.001. Moreover, the number of viable embryos were lower in the PPOS group than the ultra-long GnRHa group and GnRHant group: 2.0 (1.0-2.0) versus 4.0 (2.0-5.8) versus 2.0 (2.0-3.0), *P*<0.001.

**Table 2:** Stimulation cycle characteristics of the patients in the three groups.

	PPOS	Ultra-long GnRHa	GnRHant	*P*
Days of stimulation	10.0 (8.0-11.0)^a^	11.0 (10.0-12.0)^a^	9.0 (8.0-11.0)	<0.001
Total of gonadotropins dose	2662.5 (2100.0-3300.0)^a^	2700.0 (2250.0-3440.6)^a^	2400.0 (1846.9-3075.0)	<0.001
Fertilization method, n (%)				0.084
IVF	76 (79.2%)	201 (83.1%)	216 (80.9%)	
ICSI	15 (15.6%)	36 (14.9%)	49 (18.4%)	
IVF and ICSI	5 (5.2%)^b^	5 (2.1%)	2 (0.7%)^b^	
Cancellation rate, %	14 (14.6%)	22 (9.1%)	27 (10.1%)	0.322
Preovulation, %	1 (1.0%)	1 (0.4%)	2 (0.7%)	0.813
No. of oocytes retrieved	4.0 (2.8-7.0)^a^	11.0 (6.0-19.0)^a^	4.0 (2.5-7.0)	<0.001
No. of viable embryos	2.0 (1.0-2.0)^ab^	4.0 (2.0-5.8)^a^	2.0 (2.0-3.0)^b^	<0.001

Continuous data was presented as median (25th percentile-75th percentile) (non-normally distributed). The Kruskal-Wllis H test with Bonferroni’s adjustment was used for the comparisons of continuous variables among groups. Categorical variables were presented as n (%) and calculated by Pearson’s chi square test or Fisher’s exact test as appropriate.

PPOS, progestin-primed ovarian stimulation; GnRHa, gonadotrophin-releasing hormone agonist; GnRHant, gonadotrophin-releasing hormone antagonist; IVF, in vitro fertilization; ICSI, intra-cytoplasmic sperm injection.

a Comparison between PPOS and GnRHa, P < 0.05.

b Comparison between PPOS and GnRHant, P < 0.05.

**Table 3** showed the overall reproductive outcomes of all ET cycles originating from the three COS protocols. The thickness of endometrium in the PPOS group was lower than the ultra-long GnRHa group but similar to the GnRHant group: 10.0 (9.0-11.0) versus 11.0 (9.0-12.0) versus 10.0 (9.0-11.0), *P*= 0.015. In addition, the number and stage of embryos transferred were different among the three groups. There were 70 ET cycles in the PPOS group. Of them, 22 cycles resulted in clinical pregnancy, 5 cycles resulted in miscarriage and 17 cycles resulted in live birth. In the ultra-long GnRHa group, 207 patients completed their first ET cycles. Of them, 114 resulted in clinical pregnancy, 14 cycles resulted in early miscarriage, 1 cycle resulted in late miscarriage. In addition, 2 patients were diagnosed as ectopic pregnancy and laparoscopic surgeries were performed. At the end, 98 cycles resulted in ongoing pregnancy and 94 cycles resulted in live birth. In the GnRHant group, 215 patients completed their first ET cycles. Of them, 78 cycles resulted in clinical pregnancy, 15 cycles resulted in miscarriage. There were 2 cycles were diagnosed as ectopic pregnancy and 1 cycle resulted in stillbirth. At the end, 66 cycles resulted in ongoing pregnancy and 59 cycles resulted in live birth. 3 patients in the ultra-long GnRHa protocol and 1 patients in the GnRHant protocol did medically induced abortion because of fetal malformations.

**Table 3:** Pregnancy outcomes of patients’ first embryo transfer cycles originating from the three COS protocols.

	PPOS	Ultra-long GnRHa	GnRHant	*P*
No. of ET cycles	70	207	215	
Fresh ET cycles	0	70	71	
FET cycles	70	137	144	
Thickness of endometrium (mm)	10.0 (9.0-11.0)^a^	11.0 (9.0-12.0)^a^	10.0 (9.0-11.0)	0.015
Stage of embryo, n (%)				0.001
Cleavage embryo	34 (48.6%)^ab^	148 (71.5%)^a^	150 (69.8%)^b^	
Blastocyst	36 (51.4%)^ab^	59 (28.5%)^a^	65 (30.2%)^b^	
No. of embryos transferred, n (%)				0.006
1	11 (15.7%)^a^	11 (5.3%)^a^	29 (13.5%)	
2	59 (84.3%)^a^	196 (94.7%)^a^	186 (86.5%)	
Reproductive outcomes				
Implantation rate, n (%)	30 (23.8%)^a^	158 (39.2%)^a^	100 (24.9%)	<0.001
Biochemical pregnancy, n (%)	23 (32.9%)^a^	120 (58.0%)^a^	87 (40.5%)	<0.001
Clinical pregnancy, n (%)	22 (31.4%)^a^	114 (55.1%)^a^	78 (36.3%)	<0.001
Early miscarriage, n (%)	2 (9.1%)	14 (12.3%)	10 (12.8%)	0.892
Ongoing miscarriage, n (%)	20 (28.6%)^a^	98 (47.3%)^a^	66 (30.7%)	0.001
Late miscarriage, n (%)	3 (13.6%)^a^	1 (0.9%)^a^	5 (6.4%)	0.011
Ectopic pregnancy, n (%)	0	2(1.0%)	2(0.9%)	–
Live birth, n (%)	17 (24.3%)^a^	94 (45.4%)^a^	59 (27.4%)	<0.001
Induced abortion	0	3 (2.6%)	1 (1.3%)	–
Stillbirth	0	0	1 (1.3%)	–

Categorical variables were presented as n (%), continuous data was presented as mean± (SD). For comparison among groups, Pearson’s chi square test or Fisher’s exact test was used for categorical variables as appropriate. ANOVA with the post-hoc Bonferroni test were used for continuous variables.

PPOS, progestin-primed ovarian stimulation; GnRHa, gonadotrophin-releasing hormone agonist; GnRHant, gonadotrophin-releasing hormone antagonist; ET, embryo transfer; FET, Frozen/thawed embryo transfer; IVF, in vitro fertilization; ICSI, Intracytoplasmic sperm injection; HRT, hormone replacement therapy.

a Comparison between PPOS and GnRHa, P < 0.05.

b Comparison between PPOS and GnRHant, P < 0.05.

The implantation rate in PPOS group was significantly lower than the ultra-long GnRHa group (23.8% versus 39.2%, *P*<0.05), but similar with the GnRHant group (23.8% versus % 24.9%, *P*>0.05). In addition, based on the reproductive outcomes of first ET cycles, the PPOS protocol showed lower biochemical pregnancy, clinical pregnancy and ongoing pregnancy than the ultra-long GnRHa protocol (32.9% versus 58.0%, 31.4% versus 55.1%, 28.6% versus 47.3%, respectively). Nevertheless, biochemical pregnancy, clinical pregnancy and ongoing pregnancy showed no difference between PPOS group and GnRHant group (32.9% versus 40.5%, 31.4% versus 36.3%, 28.6% versus 30.7%, respectively). Moreover, the early miscarriage rate was similar among the three groups (9.1% versus 12.3% versus 12.8%, *P*=0.892). However, the rate of late miscarriage was higher in the PPOS group than the ultra-long GnRHa group but similar to the GnRHant group (13.6% versus 0.9% versus 6.4%, *P*=0.011). Remarkably, the live birth rate was lower in the PPOS group than the ultra-long GnRHa group (24.3% versus 45.4%, *P*<0.05) but similar to the GnRHant group (24.3% versus 27.4%, *P*>0.05).

### PPOS Protocol Versus Ultra-Long GnRHa Protocol on Reproductive Outcomes of the First ET Cycles

**Figure 2:**
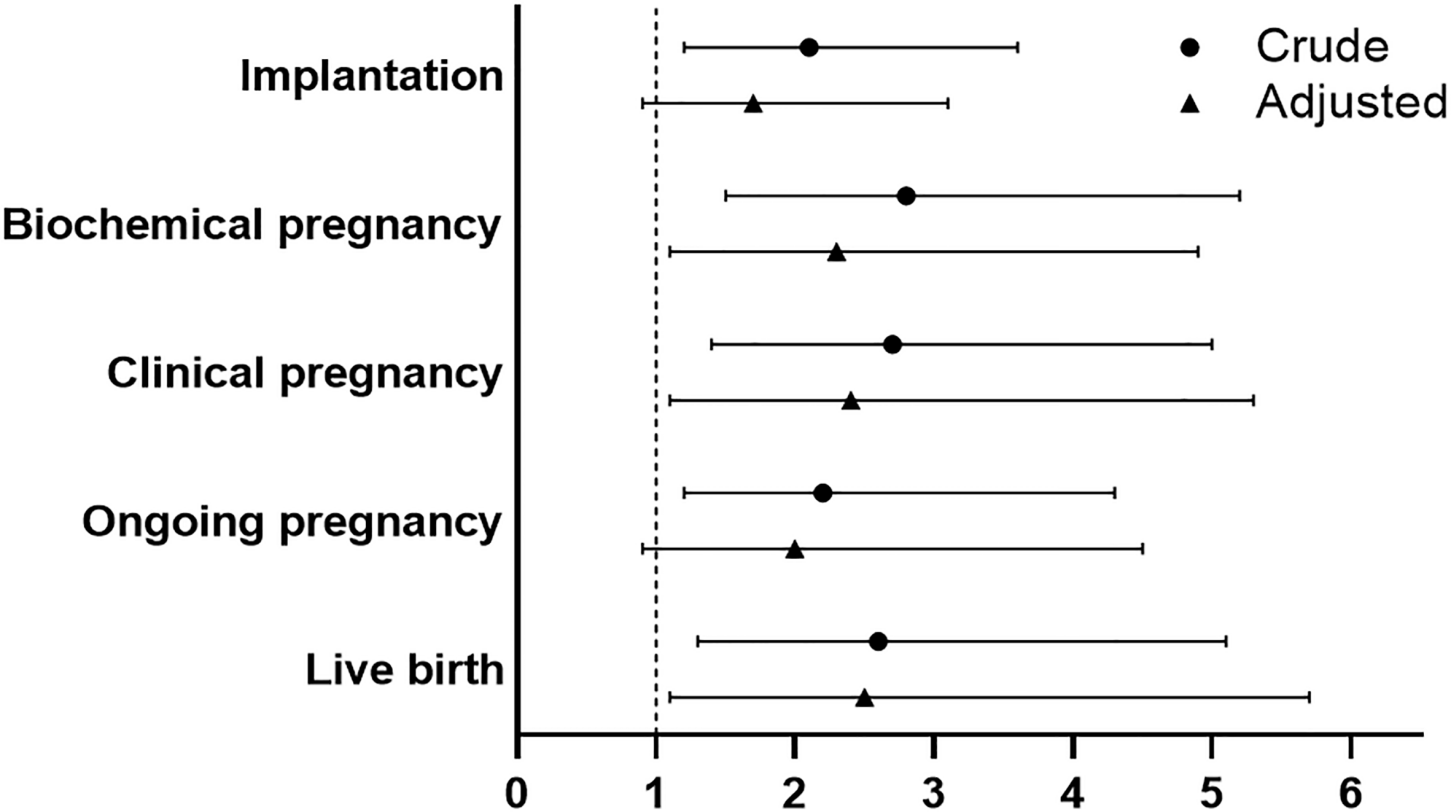
Comparison of reproductive outcomes between PPOS and ultra-long GnRHa protocol. PPOS, progestin-primed ovarian stimulation; GnRHa, gonadotrophin-releasing hormone agonist; OR, odds ratio; CI, confidence interval. The reference was the PPOS group, adjusted for maternal age, maternal BMI, total antral follicle count, adenomyosis, number of transferred embryos (1 versus 2), stage of embryo, fertilization method.

Crude and multivariable logistic regression models were used to determine the effect of PPOS versus ultra-long GnRHa protocol on the reproductive outcomes. Compared with ultra-long GnRHa protocol, PPOS protocol showed lower probability of implantation in the crude analysis (OR 2.1, 95%CI 1.2-3.6). However, there was no significant difference after adjustment in multivariable logistic regression analysis (OR 1.7, 95%CI 0.9-3.1). However, ultra-long GnRHa protocol demonstrated higher biochemical pregnancy and clinical pregnancy than PPOS protocol after adjusted for the confounding factors (OR 2.3, 95%CI 1.1-4.9, OR 2.4, 95%CI 1.1-5.3, respectively). Notably, no significant difference in ongoing pregnancy was found between PPOS protocol and ultra-long GnRHa protocol (OR 2.0, 95%CI 0.9-4.5). Additionally, PPOS protocol was associated with lower live birth rate either in crude or multivariable logistic regression analysis (OR 2.6, 95%CI 1.3-5.1, OR 2.5, 95%CI 1.1-5.7, respectively) (**Figure 2** and **Supplementary Table S2**).

### PPOS Protocol Versus GnRHant Protocol on Reproductive Outcomes of the First ET Cycles

**Figure 3:**
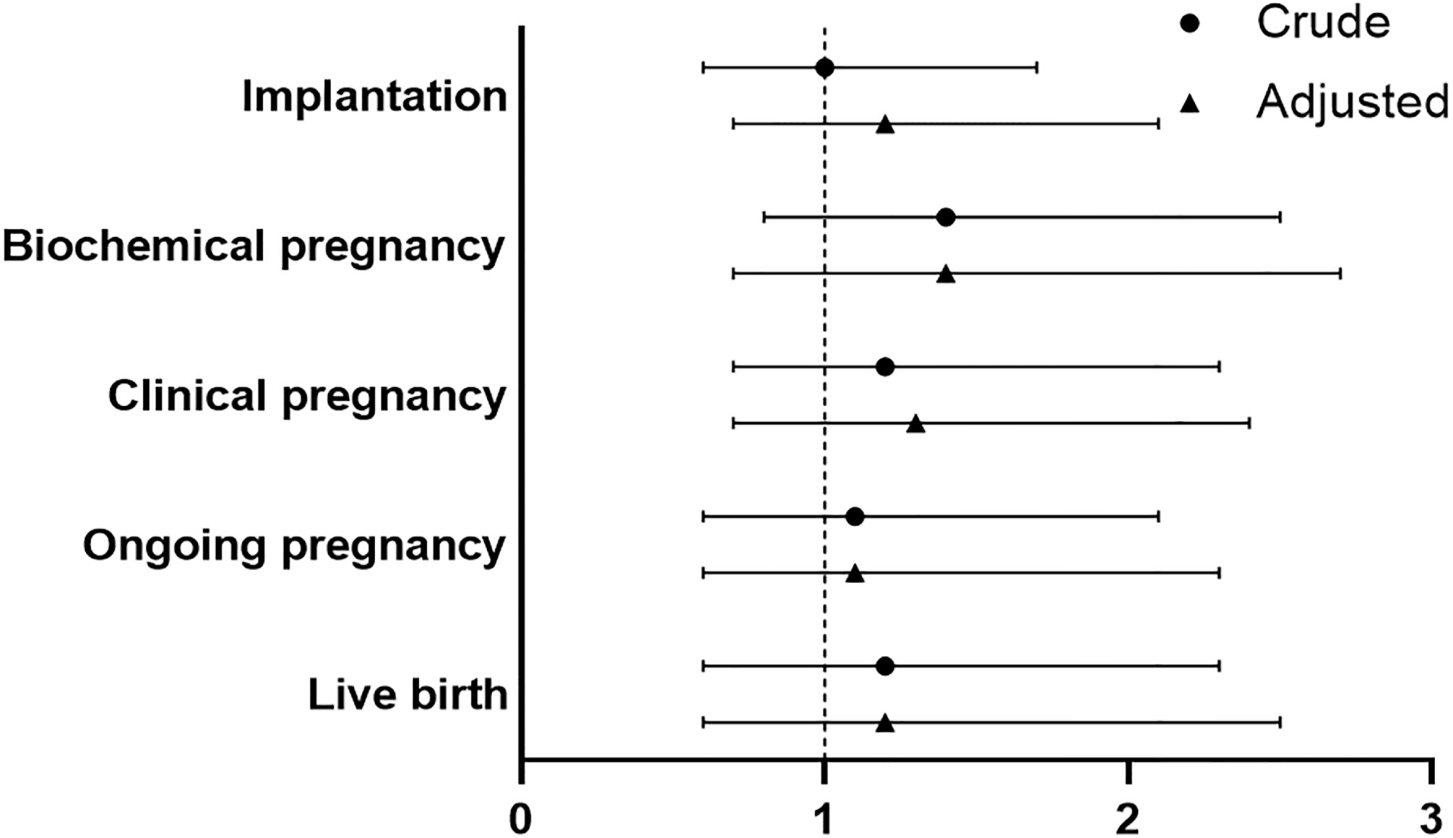
Comparison of reproductive outcomes between PPOS and GnRHant protocol. PPOS, progestin-primed ovarian stimulation; GnRHant, gonadotrophin-releasing hormone antagonist; OR, odds ratio; CI, confidence interval. The reference was the PPOS group, adjusted for maternal age, maternal BMI, total antral follicle count, adenomyosis, number of transferred embryos (1 versus 2), stage of embryo, fertilization method.

No significant difference was found between PPOS and GnRHant protocol in implantation rate for patients with endometrioma (OR 1.2, 95%CI 0.7-2.1). Moreover, PPOS protocol and GnRHant protocol showed no statistical difference in terms of biochemical pregnancy and clinical pregnancy (OR 1.4, 95%CI 0.7-2.7, OR 1.3, 95%CI 0.7-2.4, respectively). In addition, there was no significant difference in ongoing pregnancy and live birth between PPOS group and GnRHant group in multivariable logistic analysis after adjustment (OR 1.1, 95%CI 0.6-2.3, OR 1.2, 95%CI 0.6-2.5, respectively) (**Figure 3** and **Supplementary Table S3**). The effect of variables on live birth were shown in the **Supplementary Table S3**).

## Disscusion

PPOS protocol was first reported by Dr Yanping Kuang in 2015 (24). From then on, PPOS protocol has been proved to be effective in patients with various infertility conditions (25–28). Furthermore, there was general agreement that PPOS protocol could be more advantageous in planned freeze-all cycles, such as oocyte donation (29), PGT (30) and fertility preservation (31). However, to the best of our knowledge, sparse publications focused on the efficacy of PPOS regimens in patients with endometriosis, especially endometrioma. The current study dealt with a group of patients with the diagnosis of endometrioma. Our results demonstrated inferior clinical pregnancy and live birth in the cohort of PPOS protocol compared with ultra-long GnRHa protocol. In addition, there was no significant difference in terms of clinical pregnancy, ongoing pregnancy and live birth between PPOS protocol and GnRHant protocol in patients with endometrioma.

Progestins could inhibit cell proliferation, the expression of inflammatory factors, neurogenesis and neovascularization in endometriosis (32). It has been proposed that co-administration of progestins in COS might improve the quality of the oocytes and embryos. A non-inferiority randomized controlled trial has shown the effectiveness of PPOS protocol in patients with advanced endometriosis but normal ovarian reserve (33). Moreover, neonatal outcomes and congenital malformations were comparable in PPOS protocol compared with conventional ovarian stimulation protocol in maternal endometriosis (34). On the other hand, the European Society for Human Reproduction and Embryology guideline suggested that ultra-long GnRHa protocol could improve the pregnancy rate in women with moderate to advanced endometriosis in 2005 (35). In addition, the result of a cochrane systematic review found that long-term pituitary down-regulation improved the internal reproductive environment of patients with endometriosis (36). Our findings showed better clinical pregnancy and live birth in ultra-long GnRHa protocol compared with PPOS protocol among patients with endometrioma underwent IVF/ICSI-ET. Although the study population of the current study were patients with endometrioma, regardless of ovarian reserve and stages of endometriosis, our results were consistent with most of the previous publications (35, 37). Mechanically, it was found that the expression of αvβ3 integrin in endometrium might be restored when using ultra-long protocol (38). Additionally, researches also showed that the detrimental effects of cytotoxic cytokines and oxidative stress in the ovary might be reduce when using ultra-long GnRHa protocol (39). Nevertheless, a clinically oriented review in 2021 suggested that GnRH antagonist or PPOS can be more appropriate than prolonged suppression for endometriosis (15). However, no high-quality and direct evidence about COS protocol for endometriosis was provided in this review. In consequence, for patients with endometrioma underwent IVF/ICSI-ET, whether ultra-long GnRHa protocol is better than PPOS protocol in terms of live birth rate as well as cumulative live rate should be further investigated.

In recently years, GnRHant protocol has been increasingly used due to its efficacy and flexibility. A study published in 2020 found that PPOS and GnRH antagonist protocol were equally effective in terms of oocytes retrieved for patients with endometriosis underwent fertility preservation (16). Similarly, our study showed no significant difference in clinical pregnancy, ongoing pregnancy and live birth between PPOS protocol and GnRHant protocol. However, the complexity of the disease and various presentations precludes a single COS protocol for endometriosis. Therefore, more researches are still warranted to determine the optimal COS protocol for patients with endometrioma.

One patient in the PPOS, one patient in the ultra-long GnRHa group and two patients in the GnRHant group were found to experience early ovulation. All of them only had one dominant follicle. The rate of premature ovulation in our study was not higher than that reported by previously published studies (40, 41). So, early ovulation was well avoided in the three protocols. Furthermore, the total cycle cancellation rates were similar among the three study groups.

There were several limitations in our study. Firstly, the retrospective design of the current study did not allow for proper randomization and control of confounding variables. Although multivariable logistic regression models were conducted to adjust for several potential confounders, residual confounding may exist because of unknown and not included covariates. In addition, the age and ovarian reserve in the ultra-long GnRHa group were better than the other two groups, which may bias the final conclusions and could not be eliminated by statistical correction. Secondly, we only analyzed the reproductive outcomes of the first ET cycles, but some patients may get pregnant in the substantial ET cycles. The cumulative live birth of the three studied protocols should be sustaining monitored. However, the highest quality embryos were transferred in all the patients’ first ET cycles. Thus, the outcomes of the first ET cycles could reflect the quality of oocytes, embryos and ultimately the effectiveness of COS protocol. Lastly, the small number of cases made it unfeasible to perform miscarriage or subgroup analyses and led to wide CIs in the estimates of association.

## Conclusions

Base on the reproductive outcomes of the first ET cycles, PPOS protocol showed inferior reproductive outcomes compared with ultra-long protocol in terms of clinical pregnancy and live birth for patients with endometrioma. However, there was no significant difference in clinical pregnancy, ongoing pregnancy and live birth between PPOS protocol and GnRHant protocol. The cumulative pregnancy of the studied protocols should be further monitored. In addition, prospective large randomized-controlled trials are warranted to confirm our findings and determine the effect of the three studied protocols on reproductive outcomes.

## Data Availability Statement

The raw data supporting the conclusions of this article will be made available by the authors, without undue reservation.

## Ethics Statement

The studies involving human participants were reviewed and approved by the ethics committee of the Second Hospital of Hebei Medical University. Written informed consent for participation was not required for this study in accordance with the national legislation and the institutional requirements.

## Author Contributions

A-MY and G-MH had conceived the research and made the design. A-MY, NC, LL, and T-FF did the data collection and provided statistical expertise. NC and A-MY wrote the first draft of the manuscript. A-MY and LL revised it critically for important intellectual content. WW, YH, Y-ZW, X-QZ, and Z-MZ gave statistic assistance and checked the grammar. LL, B-JS, G-MH, and NC obtained funding. All authors agreed to be accountable for the content of the work. All authors contributed to the article and approved the submitted version.

## Funding

This study was supported by Natural Science Foundation of Hebei Province (Beijing-Tianjin-Hebei Cooperation Special Project) (H2019206707), S&T Program of Hebei (20377714D, 21377720D, 21377721D), Innovation Capability Enhancement Program of Hebei Province (Hebei Clinical Medical Research Center Special Project) (20577710D). Medical Science Research Project of Hebei Province (20211494), Science Foundation project of the Second Hospital of Hebei Medical University (2HC202140).

## Conflict of Interest

The authors declare that the research was conducted in the absence of any commercial or financial relationships that could be construed as a potential conflict of interest.

## Publisher’s Note

All claims expressed in this article are solely those of the authors and do not necessarily represent those of their affiliated organizations, or those of the publisher, the editors and the reviewers. Any product that may be evaluated in this article, or claim that may be made by its manufacturer, is not guaranteed or endorsed by the publisher.
