# Supplementary material for: Progestin-Primed Ovarian Stimulation Protocol for Patients With Endometrioma
Source: Front Endocrinol (Lausanne). 2022 Apr 28;13:798434. doi: 10.3389/fendo.2022.798434 (PMC9096226; doi:10.3389/fendo.2022.798434)
Supplement: Supplementary file 1 [file Table_1.docx]

**Supplementary TABLE S1** Comparison of reproductive outcomes between PPOS protocol and ultra-long GnRHa protocol.

|  | **PPOS versus Ultra-long GnRHa** | | | |
| --- | --- | --- | --- | --- |
|  | **Crude OR (95% CI)** | ***P*** | **Adjusted OR (95% CI)** | ***P*** |
| Implantation | 2.1 (1.2, 3.6) | 0.012 | 1.7 (0.9, 3.1) | 0.109 |
| Biochemical pregnancy | 2.8 (1.5, 5.2) | 0.0009 | 2.3 (1.1, 4.9) | 0.037 |
| Clinical pregnancy | 2.7 (1.4, 5.0) | 0.0023 | 2.4 (1.1, 5.3) | 0.030 |
| Ongoing pregnancy | 2.2 (1.2, 4.3) | 0.0151 | 2.0 (0.9, 4.5) | 0.100 |
| Live birth | 2.6 (1.3, 5.1) | 0.0061 | 2.5 (1.1, 5.7) | 0.034 |

PPOS, progestin-primed ovarian stimulation; GnRHa, gonadotrophin-releasing hormone agonist; OR, odds ratio; CI, confidence interval.

*The reference was the PPOS group, adjusted for maternal age, maternal BMI, total antral follicle count, adenomyosis, number of transferred embryos (1 versus 2), stage of embryo, fertilization method.
