# Supplementary material for: Progestin-Primed Ovarian Stimulation Protocol for Patients With Endometrioma
Source: Front Endocrinol (Lausanne). 2022 Apr 28;13:798434. doi: 10.3389/fendo.2022.798434 (PMC9096226; doi:10.3389/fendo.2022.798434)
Supplement: Supplementary file 2 [file Table_2.docx]

**Supplementary TABLE S2** Comparison of reproductive outcomes between PPOS and GnRHant protocol per transfer cycle.

|  | **PPOS vs GnRHant** | | | |
| --- | --- | --- | --- | --- |
|  | **Crude OR (95% CI)** | ***P*** | **Adjusted OR (95% CI)** | ***P*** |
| Implantation | 1.0 (0.6, 1.7) | 0.960 | 1.2 (0.7, 2.1) | 0.611 |
| Biochemical pregnancy | 1.4 (0.8, 2.5) | 0.277 | 1.4 (0.7, 2.7) | 0.291 |
| Clinical pregnancy | 1.2 (0.7, 2.3) | 0.493 | 1.3 (0.7, 2.4) | 0.497 |
| Ongoing pregnancy | 1.1 (0.6, 2.1) | 0.757 | 1.1 (0.6, 2.3) | 0.722 |
| Live birth | 1.2 (0.6, 2.3) | 0.632 | 1.2 (0.6, 2.5) | 0.592 |

PPOS, progestin-primed ovarian stimulation; GnRHant, gonadotrophin-releasing hormone antagonist; OR, odds ratio; CI, confidence interval

*The reference was the PPOS group, adjusted for maternal age, maternal BMI, total antral follicle count, adenomyosis, number of transferred embryos (1 versus 2), stage of embryo, fertilization method.
