# Supplementary material for: Progestin-Primed Ovarian Stimulation Protocol for Patients With Endometrioma
Source: Front Endocrinol (Lausanne). 2022 Apr 28;13:798434. doi: 10.3389/fendo.2022.798434 (PMC9096226; doi:10.3389/fendo.2022.798434)
Supplement: Supplementary file 3 [file Table_3.docx]

**Supplementary TABLE S3** The effect of variables on live birth in multivariate logistic regression analysis in the study

| Variables | **Live birth** | |
| --- | --- | --- |
|  | **OR (95% CI)** | ***P*** |
| Maternal age | 0.9 (0.8, 0.9) | <0.001 |
| Maternal BMI | 1.0 (0.9, 1.0) | 0.330 |
| Total AFC | 1.0 (0.9, 1.0) | 0.836 |
| Fertilization method |  |  |
| IVF | Reference |  |
| ICSI | 0.9 (0.5, 1.8) | 0.814 |
| IVF/ICSI | 2.1 (0.5, 9.8) | 0.339 |
| Adenomyosis | 1.3 (0.6, 2.8) | 0.552 |
| COS protocol |  |  |
| PPOS | Reference |  |
| Ultra-long GnRHa | 2.5 (1.1, 5.7) | 0.034 |
| GnRHant | 1.2 (0.6, 2.5) | 0.592 |
| No. of embryos transferred |  |  |
| 1 | Reference |  |
| 2 | 1.4 (0.4, 2.4) | 0.961 |
| Stages of embryo |  |  |
| Cleavage embryo | Reference |  |
| Blastocyst | 1.2 (0.7, 2.0) | 0.533 |

Multivariable logistic regression analysis was used to determine the independent effect of variables on live birth.

OR, odds ratio; CI, confidence interval; BMI, body mass index; AFC, antral follicle count; IVF, in vitro fertilization; ICSI, Intracytoplasmic sperm injection; COS, controlled ovarian stimulation; PPOS, progestin-primed ovarian stimulation; GnRHa, gonadotrophin-releasing hormone agonist; GnRHant, gonadotrophin-releasing hormone antagonist.
